# 3D Direct Printing of Silicone Meniscus Implant Using a Novel Heat-Cured Extrusion-Based Printer

**DOI:** 10.3390/polym12051031

**Published:** 2020-05-01

**Authors:** Eric Luis, Houwen Matthew Pan, Swee Leong Sing, Ram Bajpai, Juha Song, Wai Yee Yeong

**Affiliations:** 1 Singapore Centre for 3D Printing, School of Mechanical and Aerospace Engineering, Nanyang Technological University, 50 Nanyang Avenue, Singapore 639798, Singapore; G001@ntu.edu.sg (E.L.); slsing@ntu.edu.sg (S.L.S.); 2 School of Chemical and Biomedical Engineering, Nanyang Technological University, 70 Nanyang Avenue, Singapore 639798, Singapore; matthew.pan@u.nus.edu (H.M.P.); songjuha@ntu.edu.sg (J.S.); 3 Center for Population Health Sciences, Lee Kong Chian School of Medicine, Nanyang Technological University, 11 Mandalay Road, Singapore 308232, Singapore; r.bajpai@keele.ac.uk; 4 School of Primary, Community and Social Care, Keele University, Keele ST5 5BG, UK

**Keywords:** 3D printing, additive manufacturing, material extrusion, silicone, meniscus implant

## Abstract

The first successful direct 3D printing, or additive manufacturing (AM), of heat-cured silicone meniscal implants, using biocompatible and bio-implantable silicone resins is reported. Silicone implants have conventionally been manufactured by indirect silicone casting and molding methods which are expensive and time-consuming. A novel custom-made heat-curing extrusion-based silicone 3D printer which is capable of directly 3D printing medical silicone implants is introduced. The rheological study of silicone resins and the optimization of critical process parameters are described in detail. The surface and cross-sectional morphologies of the printed silicone meniscus implant were also included. A time-lapsed simulation study of the heated silicone resin within the nozzle using computational fluid dynamics (CFD) was done and the results obtained closely resembled real time 3D printing. Solidworks one-convection model simulation, when compared to the on-off model, more closely correlated with the actual probed temperature. Finally, comparative mechanical study between 3D printed and heat-molded meniscus is conducted. The novel 3D printing process opens up the opportunities for rapid 3D printing of various customizable medical silicone implants and devices for patients and fills the current gap in the additive manufacturing industry.

## 1. Introduction

The meniscus, which is a pair fibrocartilaginous cushion within the knee joint, acts as a weight bearing cushion, lubricating device and knee stabilizer. It is mainly C-shaped and triangular shaped in the horizontal and cross-sectional sections, respectively. Its relatively centralized acellular extracellular matrix and limited peripheral vascular supply from the meniscocapsular plexus severely restricts the meniscus regenerative capability when damaged or torn. The compressive modulus of 0.03 MPa is weakest at its supero-medial portion of the posterior horns and these regions are the commonest sites of tear and injury [1].

Treatment options for meniscus tears include direct repair, replacement with either scaffolds or implants and partial or total meniscectomy (removal of the damaged meniscus tissues). Repair methods for meniscal tears with sutures, pins and fibrin glue were used with limited success due to poor vascular supply. Replacement with meniscus scaffolds made of polycaprolactone (PCL) or collagen have produced equivocal results owing to inferior compressive mechanical properties. Partial meniscus implants which include collagen meniscus implant (Menaflex) and Actfit were used with limited success and have not yet gained approval from FDA. Total meniscus implant NuSurface is the only total meniscus implant currently undergoing Sun Clinical Trial 2 [2,3].

Silicone is used in biomedical manufacturing for biliary stents [4], cochlear implants [5], peripheral nerve sheaths [6], breast [7,8,9,10], chin, nose [11], testicular and hand implants [12], interphalangeal joint replacement implants [13], knee prosthesis [14], cardiopulmonary bypass tubing [15] and silicone membranes [16]. Recently, microfluidics [17], lab-on-a-chip [18] and biosensors [19] have also been manufactured with silicone. Despite these, the manufacturing of meniscus implants using silicone was limited. Meanwhile, most information in literature are on the general properties of silicone resins but little on the rheological properties. Takahashi et al. studied the rheological behavior of several silicone resins in the un-crosslinked state and found that the temperature dependence of the viscoelastic behavior can be described by a Williams–Landel–Ferry (WLF) equation [20].

Most of the implants are produced by indirect molding technique, whereby the mold is first 3D printed or machined and the silicone is subsequently poured into the mold and left to cure. This indirect molding technique, both time consuming and costly, also posed technical challenges when customized implants of different geometries or hollow silicone parts need to be manufactured.

Industrial silicone 3D printing began in 2015. The current state-of-the art in silicone 3D printing includes (a) using UV light of 365 nm to cure UV-sensitive silicone, as they are being deposited layer-by-layer by an inkjet printer (ACEO, Wacker, Munich, Germany), (b) freeform reversible embedding technique using a 2-part A/B silicone where the part A-silicone catalyst-cross-linker is extruded into a bath of part B-silicone (Fripp Design, Sheffield, UK) [21], (c) using a progressive cavity pump to extrude moisture-cured one part Oxime silicone elastomer [22], (d) a hybrid ink-jetting and UV-extrusion techniques with a printing speed which is 20 times faster [23], (e) a combination stereolithographic with low-one-photon-polymerization (LOPP) [24] and (f) 3D printing of PDMS elastomer in a hydrophilic Carbapol bath support [25].

The first successful direct 3D printing of heat-cured medical grade silicone meniscus implants is reported in this study. To date, no direct 3D printing of medical silicone implants has been described. First, optimum curing temperatures and times are obtained from the rheological characterization of the silicone resins [26,27]. This is followed by parametric optimization of nozzle diameters, nozzle temperatures and bed temperatures, using a range of parametric values, to print out standard ASTM cylindrical blocks, T-bones and finally meniscus implants. Computational fluid dynamics (CFD) simulation is used to study the heat transfer within the silicone resin across the heated nozzle and Solidworks is used to study the heat gradient distribution across the silicone meniscus implant. The results were validated against experimental values using standard heat probes. The new direct 3D printing technology provides an avenue for rapid 3D printing of various customizable medical silicone implants and devices for patients and fills the current gap in the additive manufacturing (AM) industry.

## 2. Materials and Methods

### 2.1. Silicone Material Specification

The silicone material used in this study is a water-white translucent, two-part platinum-catalyzed silicone elastomer that cure upon exposure to room temperature or can be accelerated by heat (Smooth-On®, Smooth-On Inc, Macungie, PA, USA). Ecoflex30 and Ecoflex50 are chosen for its safety and biocompatible profiles. They are mixed 1A:1B by weight or volume and cured at room temperature with negligible shrinkage. Their low viscosities permit easy mixing and de-airing. The properties of Ecoflex30 and Ecoflex50 are shown in Table 1.

### 2.2. Experimental Setup

**Figure 1:**
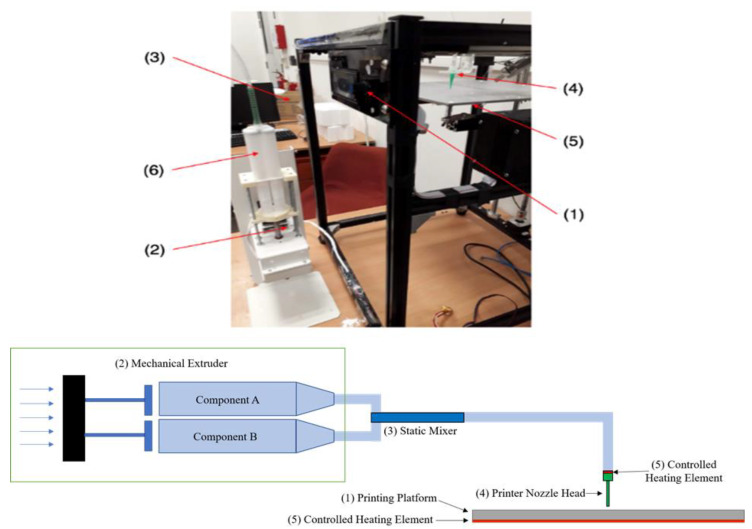
Experimental setup for heat cure extrusion-based additive manufacturing (AM): legends: (1) motion control platform, (2) extruder, (3) static mixer, (4) printhead, (5) heating elements.

The system consists of six key components: (1) a motion control platform based on an open-source 3D printer (CoreXY, Guangzhou, China), (2) a syringe-pump extruder with accuracy of 0.05 μm to dispense the material, (3) a double barrel syringe connected to a static mixer using tubing (TIUB05C, SMC Corporation, Tokyo, Japan), (4) stainless nozzles with tip size between 20 and 21 gauges (inner diameter from 0.5 mm to 0.6 mm), (5) heating elements on the printer nozzles and platform with their controllers, as shown in Figure 1.

Parts A and B of the Ecoflex ^®^ resins were added into the double barrel syringe with mix ratio of 1:1 by volume prior to fabrication. The resins were subsequently passed through the static mixer before extrusion. 3D printing software (Repetier-Host, Hot-World GmbH & Co. KG, Willich, Germany) was used to control the 3D printer while Slic3r was used for tool path generation.

### 2.3. Process Parameters and Experiment Design

In this study, several key process parameters, such as the solution flow rate (*Q*), nozzle-to-substrate distance (*h*), were fixed to simplify the optimization process. Before the 3D printing of meniscus implants, several experiments were conducted to find a suitable range of the process parameters. The analysis of the 3D printed meniscus fabricated under varied temperature of the heating elements was done to find out the best heat cured meniscus.

For the first part, inner nozzle diameter (*d*) and the print speed (*v*) were analyzed through fabricating several cylinders and T-bones. Meanwhile, checks were conducted on the layer-layer deformation and whether there is void inside the specimens. Specimens with complicated structure were fabricated to verify whether these ranges of the process parameters are feasible for the meniscus fabrication. In the second part, several printing experiments were conducted to find out the optimized combination of the temperatures of the heated platform (*T*_1_) and the heating elements on the print head (*T*_2_). Lastly, comparison between the compressive properties of the 3D printed meniscus and heat molded meniscus was done.

### 2.4. Rheology Test

Rheology test was conducted to measure the gelation in silicone since it is instantaneous using a TA Texas Instruments DHR2 rheometer (New Castle, DE, USA). The testing geometry used is a 40 mm steel Peltier parallel plate which is fitted after instrument inertia calibration, bearing friction correction and geometry inertia calibration. The rheometer was operated in parallel-plate mode.

For the experiments, the two components Parts A and B of each silicone resin Ecoflex30 and Ecoflex50 (cross-linker with catalyst and polymer) were manually mixed according to the manufacturer’s datasheet. Characterization of the rheological behavior of the silicone in triplicates was done in both steady and oscillation modes at different isothermal temperatures (30 °C to 80 °C) according to DIN 53529 which is similar to ASTM D 5289. The selected temperatures were controlled by the Peltier device with a cooling pump at an accuracy of ±0.1 °C. For the dynamic oscillatory rheology investigation, the samples were exposed to increasing strain rate (0.00 to 3.00 s^−1^) at a constant frequency (1 Hz) to determine the linear viscoelastic range of the samples [28].

The curing times, complex viscosities and complex moduli of the silicone at various temperature are determined using the temperature ramp mode from 30 °C to 80 °C with ramp rate of 5 °C/min. A normal force control of 2 N, with tolerance of 1.75 N is applied on the torsion geometry with gap change limit maintained at 550 μm. The control strain is set at 0.025% and constant angular frequency was set at 3 rad/s. The data are collected and plots of G’, G’’ and tan (delta) against temperature and time are performed [29].

The frequency sweep mode is used to determine the viscoelastic properties of the silicone resin in steady shear and dynamic-mechanical experiments in a frequency range between 0.01 and 100 rad/s. The heat-treated material was first ground and subsequently pressed to dense discs at 70 °C like the untreated material. Before being measured, all samples were dried in a desiccator at room temperature for 12 h.

### 2.5. Surface Morphology of 3D Printed Silicone Meniscus Implant

Nikon SMZ 1000 (Tokyo, Japan) was used for 6× magnification surface visualization of the overall layer-by-layer step morphology of the 3D printed implants and Eclipse LV 150 (Nikon Metrology NV, Leuven, Belgium), was used for 50× magnification cross-sectional visualization of the 3D printed implants.

### 2.6. Statistical Analysis

Descriptive statistics (mean ± standard deviation) for all quantitative variables are obtained. Normality of continuous variables were tested by Kolmogorov–Smirnov and Shapiro–Wilk tests. An exploratory analysis of the correlation between meniscus length and width with nozzle and print bed temperatures using Spearman rank correlation due to small sample sizes is done. The univariate linear regression is used to evaluate the effect of nozzle and print bed temperatures on the meniscus length and width. Statistical analyses were carried out using Stata version 14.2 (StataCorp, College Station, TX, USA).

### 2.7. Computational Fluid Dynamics (CFD) Simulation Studies for Heated Printer Nozzle

The nozzle design and schematic is shown in Supporting Information, Figure S1. Using Autodesk computational fluid dynamics (CFD) 2017 software (Autodesk Inc, San Rafael, CA, USA), the input parameters for materials used are provided in Supporting Information, Table S1.

After applying the material properties, it is necessary to provide the boundary conditions. The flow simulation works only in a closed region. Thus, there is a need to create a computational domain and a fluid sub domain [30]. Boundary conditions are set as follows: Surface Temperature—60 °C, Pressure—0 Pa Gage, Volume Flow Rate—50 mm^3^/min, Temperature—25 °C. 

### 2.8. Solidworks Simulation Studies for 3D Printed Silicone Meniscus Implant

Solidworks 2018 (Dassault Systems Solidworks Corporation, Waltham, MA, USA) was used to study the temperature gradient distribution in the 3D printed silicone meniscus implants. Analysis using the stimulation module can reduce the number of product development cycles and time to market (TTM), optimize the meniscus design and reduce cost by testing the model on the computer rather than physical tests. From the simulation task pane, thermal study and the material properties are first entered: Elastic modulus (0.290075 × 10^6^), Poisson’s ratio (0.47), shear modulus (0.0029 × 10^6^ Psi), mass density (143.58 lb/ft^3^), tensile strength (0.797708 ksi), compressive strength (4.35113 ksi), yield strength (0.758547 ksi), thermal expansion coefficient (540 × 10^−6^), thermal conductivity (4.77 BTU ft/h), specific heat (1.00602 BTU/lb.P). Subsequently, this is followed by automatic generation of mesh of finite elements for FEA by the program. To set the boundary conditions, the perimeter of each of the eleven layers sliced by the Slic3r program was used to estimate the amount of time taken to print each layer and the estimated perimeter in millimeters and printing time taken in seconds are expressed below: From the first layer to the final (eleventh) layer, the perimeters are 273, 274, 165.7, 151.8, 138.9, 125.8, 112.3, 97.9, 82.0, 64.0 and 43.0 mm requiring estimated printing times of 330, 330, 183, 167, 153, 138, 124, 108, 90, 70 and 47 seconds, respectively. The perimeters of each layer was used to estimate the total amount of time taken for each layer to be printed as shown in Supporting Information, Table S2. Experimental temperature values at every layer laid down are measured with heat probe model HT with K-type thermistor.

## 3. Results

### 3.1. Rheology

#### 3.1.1. Steady Shear Flow Study

**Figure 2:**
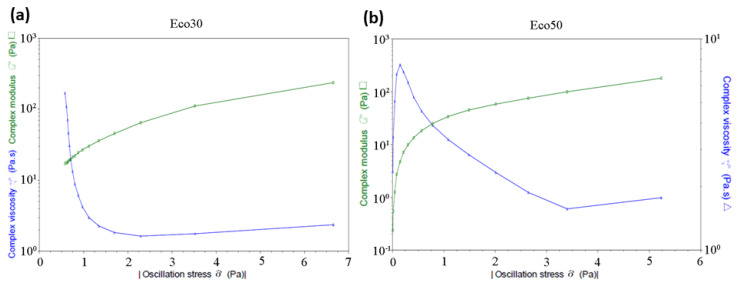
(**a**) Ecoflex30 and (**b**) Ecoflex50 complex viscosity and complex modulus versus shear stress.

The uninterrupted extrusion of silicone is crucial for the 3D printing of the meniscus implants. The extrusion is dependent on their viscous behavior under different shear rates. The rheological fingerprints of both Ecoflex30 and Ecoflex50 silicone samples at the temperature 50 °C are shown in Figure 2. Both Ecoflex30 and Ecoflex50 showed non-Newtonian behavior. The shear stress of Ecoflex30 is proportional to the shear rate at lower shear rates. Its viscosity was around 3 Pa·s, the so-called zero-shear rate viscosity *η*_0_. At shear rates of about 0.5 s^−1^, the Ecoflex30 viscosity starts to decrease significantly, until it starts to level off at higher shar rates of 3 s^−1^.

**Figure 3:**
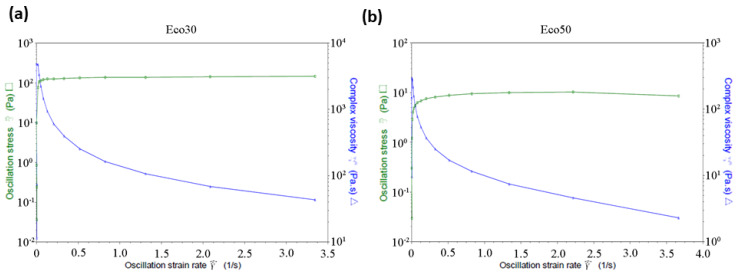
(**a**) Ecoflex30 and (**b**) Ecoflex50 complex viscosity and shear stress versus strain rate.

Similar behavior for PDMS solutions is reported [31]. Figure 3 shows a sharp decrease in the viscosity of both Ecoflex30 and Ecoflex50, from about 200 to 20 Pa·s and from 500 to 5 Pa·s, respectively, over the shear rate range 0.01 to 5 s^−1^ At very high shear rates the viscosity may again become independent of shear rates, approaching the infinite-shear rate viscosity *η_∞_*. Polymer degradation becomes a serious problem before sufficiently high shear rates can be obtained which made *η_∞_* not usually measurable. The behavior of Ecoflex30 under steady shear flow in the range above 3.5 s^−1^ is shear thinning or pseudoplastic behavior. The decreasing viscosity with increasing shear rates is utilized for the current extrusion nozzle design.

#### 3.1.2. Transient Shear Stress Response

For rheologically complex materials, understanding the transient shear behavior is important. To examine the transient behavior of Ecoflex30 and Ecoflex50, their shear stress response against time were measured. Both Ecoflex30 and Ecoflex50 show Bingham pseudoplastic shear thinning behavior on the first 250 seconds, as shown in Figure 2.

Over the tested non-Newtonian range of shear rates 0.5 < *ç*ù < 10 s^−1^, shear thinning behavior was recorded for all temperatures. The shear rate has significant effect on the flow behavior of Ecoflex30. For example at 55 °C, the viscosity of Ecoflex30 decreases from 3000 Pa·s at 0.05 s^−1^ to almost 2 Pa·s at 2.5 s^−1^ (Figure 3a) and the viscosity of Ecoflex50 decreases from 300 Pa·s at 0.05 s^−1^ to almost 2 Pa·s at 2.5 s^−1^ (Figure 3b). This effect represents a depression of 3 orders and 2 orders of magnitude, for Ecoflex30 and Ecoflex50, respectively, over a shear rate range of less than 3 s^−1^.

#### 3.1.3. Dynamic Test

The storage modulus *G*’ (elastic response) and the loss modulus *G*’’ (viscous response) are measured using a dynamic test where oscillating stresses or strains are applied to the test samples. The total resistance versus the applied strain gives the complex modulus G*.

At the start of a dynamic test, the linear viscoelastic range is defined by increasing the stress to cover a wide range. The range where complex moduli G* is constant with stress is the linear viscoelastic range which indicates that the internal bonds of the sample are still intact. The linear viscoelastic range was found to be around 35 to 50 Pa range for Ecoflex30 and 100 to 150 Pa for Ecoflex50.

**Figure 4:**
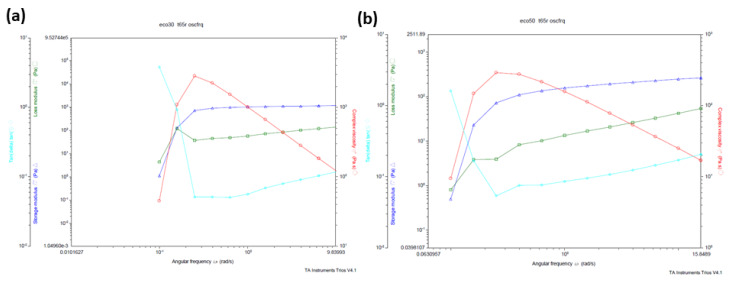
(**a**) Eco30 and (**b**) Eco50 storage modulus, loss modulus, complex viscosity vs angular frequency.

A frequency sweep test was carried out at the stress value 1.5 Pa to study the viscoelastic behavior Ecoflex30 and Ecoflex50. Both samples demonstrated elastic and viscous responses. Figure 4 show the elastic G’ and viscous modulus *G*’’ for Ecoflex30 and Ecoflex50 over the frequency range 1–100 rad/s. Ecoflex30 has both higher elastic and higher viscous modulus (Figure 4a), when compared to those of Ecoflex50 (Figure 4b). Eco30 samples also demonstrated a greater elastic response than viscous response over the entire range of frequencies.

#### 3.1.4. Gelation Times

**Figure 5:**
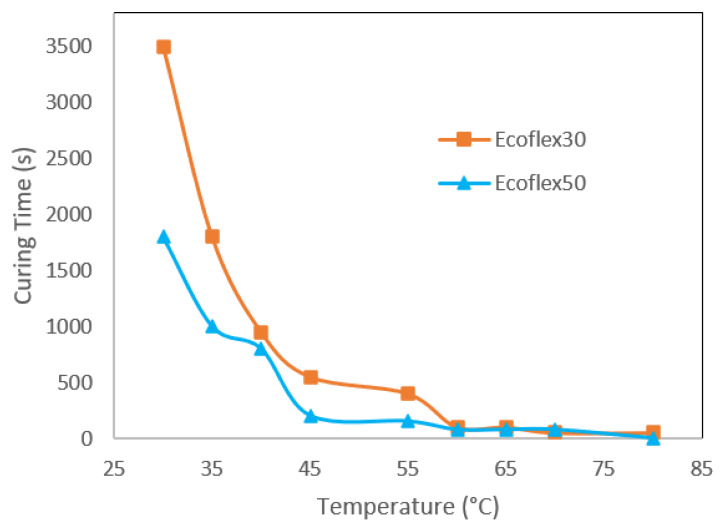
Plot of curing times (s) of Ecoflex30 (orange line) and Ecoflex50 (blue line) versus temperatures (°C).

Ecoflex 30 has a gelation time of 805 s with a crossing modulus of 130 MPa when heated to 40 °C. This is shown in Figure 5. It has a gelation time of 185 s with a crossing modulus of 365 MPa at 50 °C. With the this increase in temperature, the gelation time was shortened by 10 min, the complex modulus was also lowered by from 28 mPa to 8 mPa. However, there was an increase in complex viscosity from 5 Pa·s to over 40 Pa·s.

At below 60 °C, Ecoflex50 demonstrated longer curing times when compared to Ecoflex30. Above 60 °C, both Ecoflex30 and Ecoflex50 have similar curing times. As the temperature increases, the complex viscosity decreases according to the temperature dependence of the viscous properties of the silicone resins. However, at higher temperatures, the crosslinking rates dominate and increases the viscosity. The change in storage and loss modulus over time for Ecoflex30 and Ecoflex50 is shown in Supporting Information, Figure S2.

### 3.2. Optimization of Print Speed and Nozzle Inner Diameter

**Figure 6:**
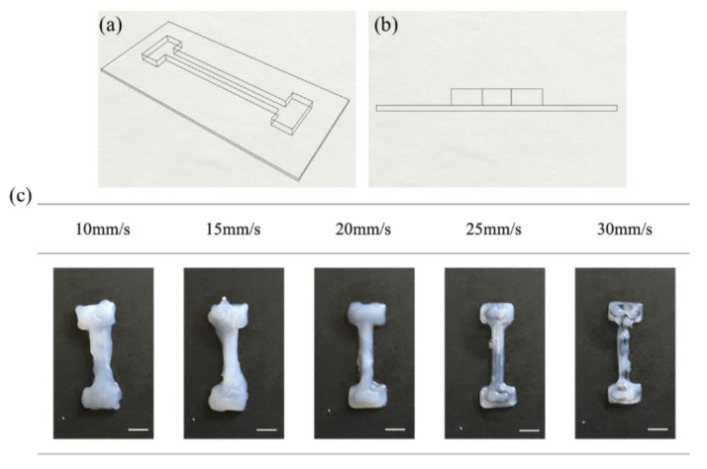
Schematic illustration of T bone: (**a**) overview and (**b**) side view. (**c**) Top view images of the 3D printed T bone under varied print speed. Scale bar: 1 cm.

An optimized combination of flow rate and print speed is critical in determining printing outcomes. In this study, all flow rates were fixed at 1.2 ml/min. An initial simple three-layer T bone was designed and printed to determine the optimized print speed. The T-bone CAD drawings, shown as Figure 6a,b, were designed with height of 3 mm, overall width of 19 mm, overall length of 55 mm and width of narrow section of 6 mm.

In this section, print speeds, varying from 10 mm/s to 30 mm/s with 5 mm/s intervals, were used to print the silicone. Other process parameters were fixed (*T*_1_ = 40 °C, *T*_2_ = 100 °C, *Q* = 1.2 ml/min and *d* = 0.90 mm) and 3 specimens were printed with each set of parameters. The top view images of selected 3D printed silicone T-bone fabricated under different print speeds were displayed in Figure 6c. With increasing print speeds, the silicone line diameter gradually decreases until it becomes spotty or discontinuous. At *v* = 30 mm/s, silicone droplets were deposited as the print speed was too high. On the other hand, a low print speed at v = 10 mm/s resulted in silicone overflow. The best printout was obtained with a print speed of 20 mm/s.

**Figure 7:**
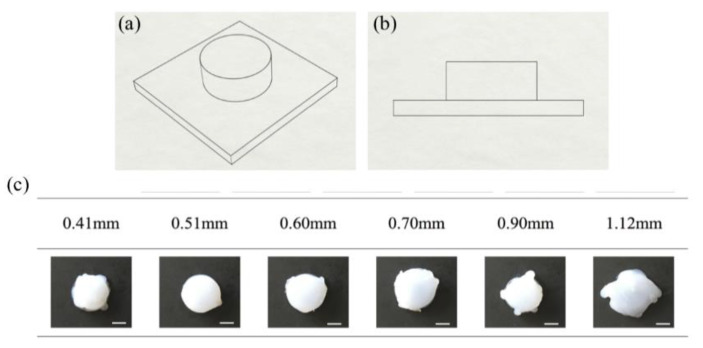
Schematic illustration of 3D printed cylinder (**a**) overview and (**b**) side view. (**c**) Cross-sectional images of the cylinder. Scale bar 1 cm.

Subsequently, cylinders were printed to determine the optimal nozzle diameter for more complex printings. The CAD drawing of the cylinders, Figure 7a,b, has a diameter of 30 mm and height of 10 mm. In this section, seven different nozzle diameters, *d,* (0.41, 0.51, 0.60, 0.70, 0.90, 1.21 mm) were used to print the cylinders. Other process parameters were fixed at *T*_1_ = 40 °C, *T*_2_ = 100 °C, *Q* = 1.2 mL/min and *v* = 20 mm/s.

Results of printed silicone cylinders are shown in Figure 7c. Using a nozzle diameter of *d* = 0.41 mm resulted in discontinuous print lines, droplets and under extrusion. Best print results, with print heights of 10 mm, were obtained with nozzle diameters of 0.51 mm and 0.6 mm, while keeping other process parameters constant. Silicone overflow due to over-extrusion were observed from *d* = 0.70 mm to *d* = 1.12 mm. Except for *d* = 0.51 mm and 0.60 mm, using all other nozzle diameters may result in under- or over-extrusion. Based on these experiments, a combination of a print speed of 20 mm/s and a nozzle inner diameter of either 0.51 mm or 0.60 mm were selected for subsequent printings. These specific process parameters will need to be modified accordingly for the printing of different specimens.

**Figure 8:**
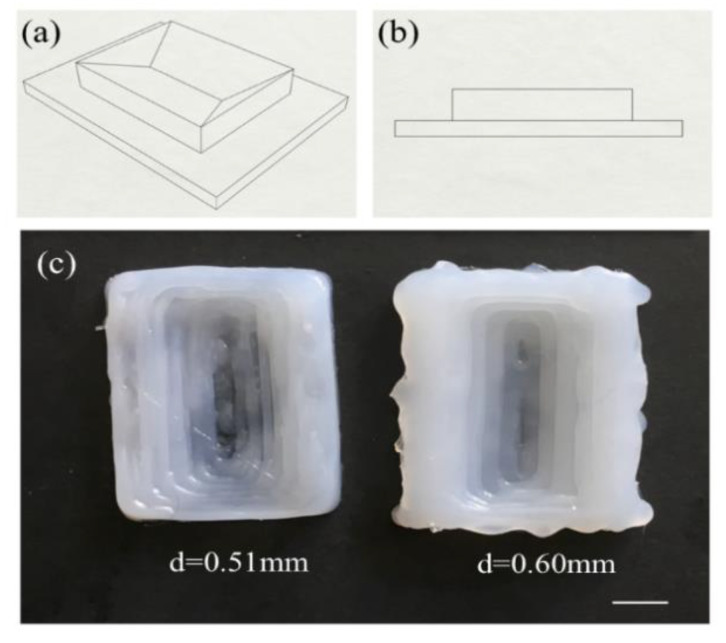
Schematic illustration of a polyhedron in (**a**) overview and (**b**) side view. (**c**) Top view images of the polyhedron. Scale bar: 1 cm.

A more complicated polyhedron with four slopes was printed. The CAD dimensions of the polyhedron are shown in Figure 8a,b, which is approximately 50 mm × 40 mm × 20 mm and 10 mm deep. After slicing, this polyhedron has 20 layers and the slope starts from the 11th layer. Figure 8c shows the top view images of the polyhedron, which fabricated based on the previous process parameters (*T*_1_ = 40 °C, *T*_2_ = 100 °C, *Q* = 1.2 ml/min, *v* = 20 mm/s, *d* = 0.51 mm and 0.61 mm). Overall, the layers can be clearly observed on both polyhedrons and the dimensions of these two were close to the designed values. However, with d = 0.60 mm, slight overflow was observed due to over-extrusion and inadequate heating. This observation demonstrates that void-less silicone polyhedron with slopes can be reliably achieved.

### 3.3. Fabrication of Silicone Meniscus

**Figure 9:**
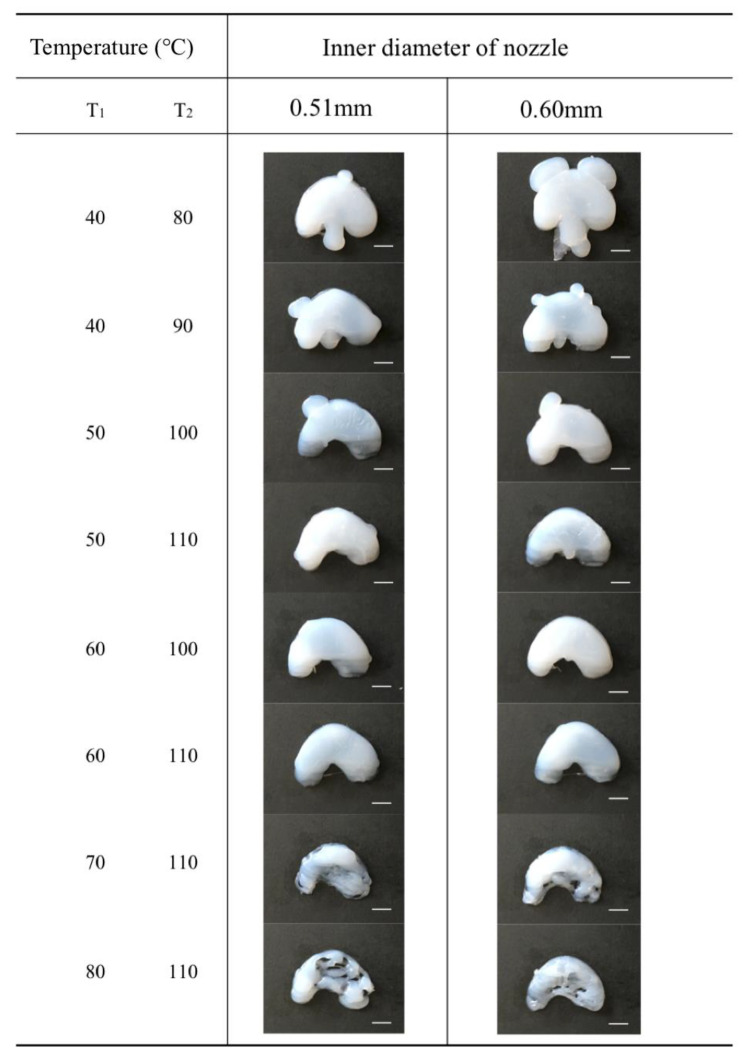
Top view images of the 3D printed silicone meniscus under varied heating temperature (*T*_1_ = nozzle temperature and *T*_2_ = platform temperature) and inner nozzle diameter. Scale bar: 1 cm.

To determine the effects of temperature variation on the silicone meniscus printing, a series of experiments were conducted. The temperature of the heated platform was varied from 80 °C to 110 °C with intervals of 10 °C and the nozzle temperature was varied from 40 °C to 80 °C with intervals of 10 °C. As shown in Figure 9, several combinations of the temperatures were selected to fabricate the silicone meniscus.

In general, a larger volume of silicone is extruded with a larger nozzle diameter. This extruded volume decreases with an increase in temperature and is caused by clogging of the nozzle or tubing. Clogging is particularly significant at nozzle temperatures of more than 70 °C and above. Nozzle temperatures of 50 °C and below resulted in inadequate heating, inadequate curing and overflow. With increasing height of meniscus being printed, heat conduction via the thickness of silicone meniscus is impaired and this may similarly result in silicone overflow and collapse of the printout. Therefore, a higher platform temperature of *T*_2_ = 100 °C to 110 °C is required. From Figure 9, a combined nozzle temperature *T*_1_ = 60 °C platform temperature *T*_2_ = 110 °C and nozzle diameter d = 0.51 mm gave the best printout of meniscal implant.

### 3.4. Association between Meniscus Length and Width with Nozzle and Print Bed Temperatures

**Figure 10:**
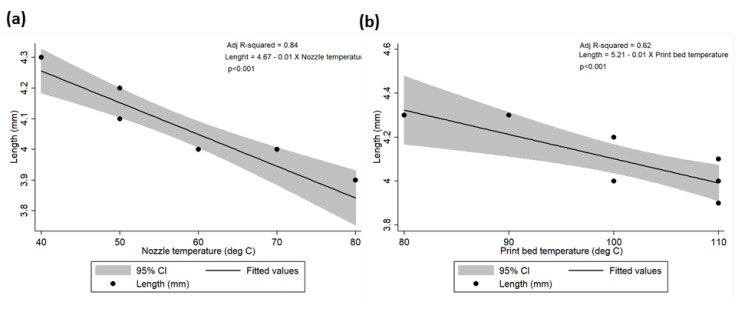
Correlation between meniscus length and (**a**) nozzle temperature and (**b**) print bed temperature.

**Figure 11:**
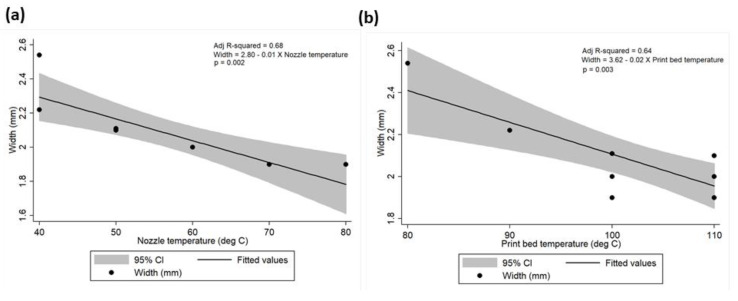
Correlation between meniscus width and (**a**) nozzle temperature and (**b**) print bed temperature.

Mean meniscus length and width were calculated to be 4.08 ± 0.14 cm (range: 3.90–4.30 cm) and 2.08 ± 0.19 cm (range: 1.90–2.54 cm), respectively. Similarly, mean nozzle and print bed temperatures were calculated as 57 ± 12.52 °C (range: 40–80 °C) and 102 ± 10.33 °C (range: 80–110 °C), respectively. A strong negative correlation has been observed between meniscus length vs. nozzle temperature (rho = −0.93; *p* < 0.01); meniscus length vs. print bed temperature (rho = −0.82; *p* < 0.01); meniscus width vs. nozzle temperature (rho = −0.84; *p* < 0.01); and meniscus width vs. print bed temperature (rho = −0.83; *p* < 0.01). The linear relationship between the meniscus length and width to the nozzle and print bed temperatures is presented in Table 2 and Figure 10 and Figure 11. The univariate regression analysis demonstrated that high amount of variability in the meniscus length and width can be explained by the nozzle and print bed temperatures independently.

### 3.5. Surface and Cross-Sectional Morphology

**Figure 12:**
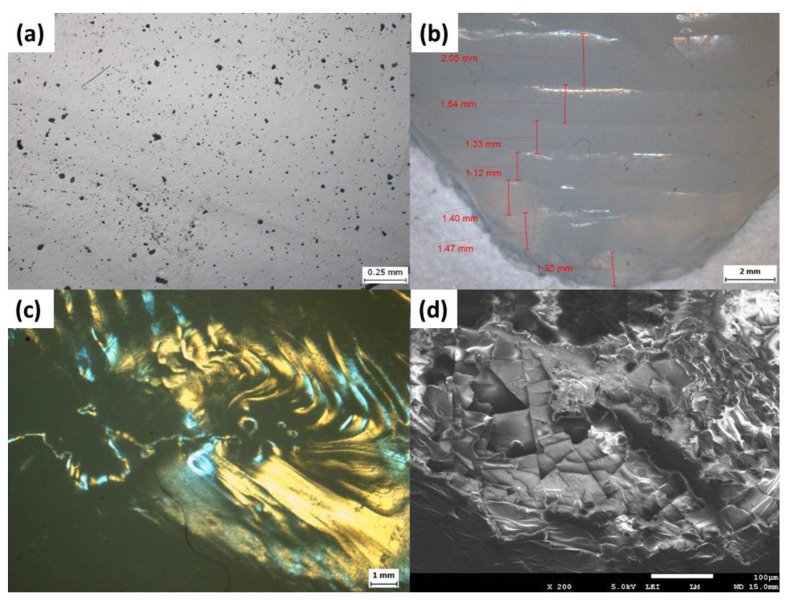
Bright field images of (**a**) surface, (**b**) posterior horn and (**c**) anterior horn of 3D printed silicone meniscus implant. (**d**) Scanning electron microscopy (SEM) image of meniscus surface at ×200 magnification.

The surface morphology observed in Figure 12a,d shows relatively smooth surface with pits measuring less than 20 micrometers due to impurities and bubbling. The orderly layer-by-layer stepwise deposition is seen in Figure 12b,c, in both the posterior and anterior horns of the meniscus, respectively. Supporting Information, Figure S3 illustrate the interlayer silicone bonding with clear lamination lines.

### 3.6. Heated Nozzle Computational Fluid Dynamics (CFD) Simulation Studies

**Figure 13:**
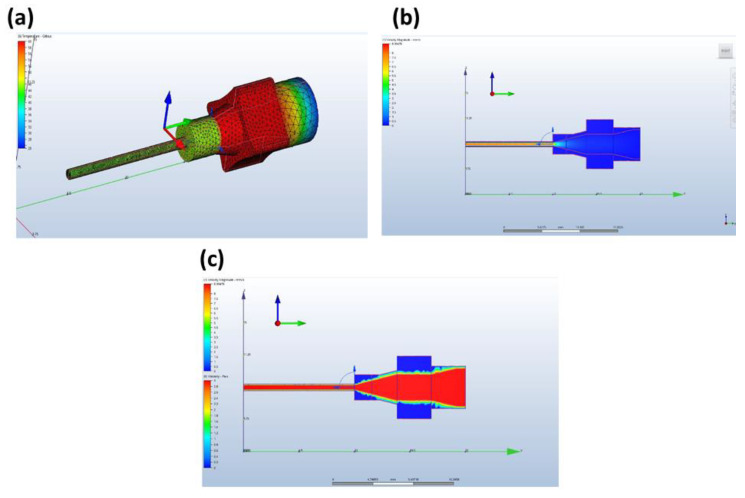
(**a**) Temperature distribution along nozzle, (**b**) velocity and (**c**) viscosity distribution of silicone resin along nozzle.

The following simulation tests results illustrate the distribution of temperature, velocity and viscosity of the silicone resin along the nozzle, as shown in Figure 13a–c below, respectively. Figure 13a shows a temperature of 60 °C at the nozzle inlet, shown in red, where it is in direct contact with the heating block. The subsequent reduction in temperature along the nozzle tip to around 40 °C is shown in dark green. These simulation temperatures correlated quite accurately with the actual temperatures of thermocouple readings of aluminum heating block thermocouples and heated platforms. Figure 13b shows a relatively slow traveling speed of silicone resin at 0.5 mm/s at the nozzle inlet, shown in blue. Within the barrel of the nozzle, the velocity of the silicone increases centripetally to about 6 mm/s, shown in yellow and 8 mm/s in the central axis, as shown in orange. Figure 13c shows a relative constant viscosity throughout the nozzle from the inlet to the outlet at 3 Pa·s, shown in red.

### 3.7. Solidworks Meniscus Implant Heat Gradient Simulation Studies

**Figure 14:**
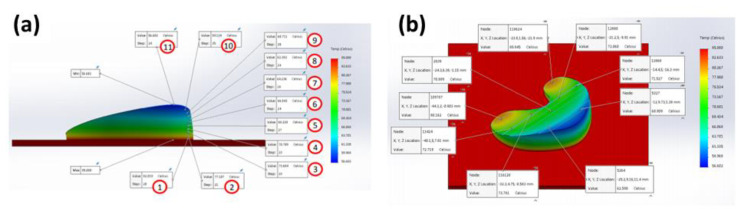
(**a**) Vertical and (**b**) horizontal temperature distribution of the 3D printed silicone meniscus implant using one convection block simulation.

**Figure 15:**
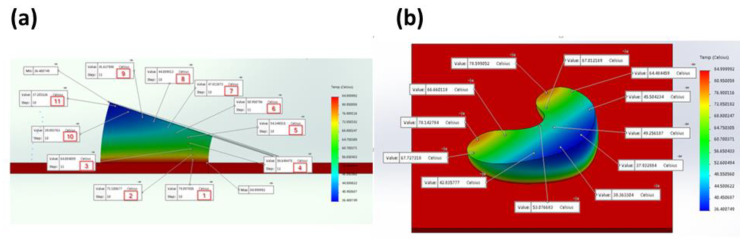
(**a**) Vertical and (**b**) horizontal temperature distribution of on-off layering simulation.

One-convection simulation and on-off layer simulation methods were performed and the results of heat gradients of printed meniscus are shown in Figure 14 and Figure 15, respectively. The simulation temperatures from one-convection model correlates more closely with the experimental results, inferring the adoption of this model for future 3D printing of implants. The comparison between thermal results using one-convection and on-off simulations is shown in Supporting Information, Figure S4.

### 3.8. Comparison of Compression Modulus of 3D Printed and Heat Molded Silicone Meniscus

**Figure 16:**
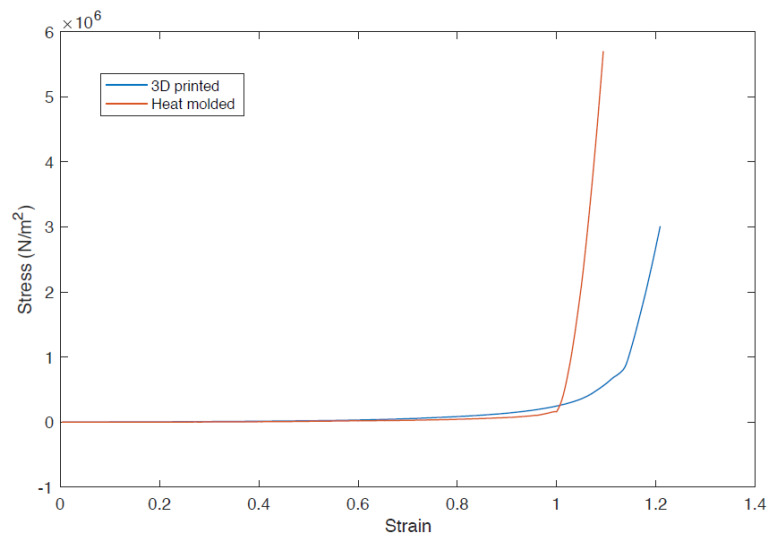
Stress vs strain plot of 3D Printed (blue line) vs heat molded silicone meniscus (red line).

The student’s independent *t*-test was used to compare the compressive modulus of the 3D printed silicone meniscus (0.838 +/− 0.070) MPa vs that of the heat-molded silicone meniscus (0.131 +/− 0.024) MPa. The 95% confidence interval of this difference range from −96,564.16 to 1665.17 MPa. There is no statistical difference between the two groups. The two-tail P is more than 0.05 (*p* = 0.058). The results demonstrate that the 3D printed silicone meniscus has similar compressive mechanical properties as that of the heat-molded silicone meniscus. Figure 16 shows the stress vs strain plot comparison of the 3D printed meniscus versus heat-molded silicone meniscus.

## 4. Discussions

This study describes the first successful direct 3D printing of heat-cure silicone meniscal implant, using biocompatible and bio-implantable silicone resins. Previous successful works with silicone extrusion, using non-heat curing technology include catalyst extrusion onto a silicone bath (Fripps, Sheffield, UK], multi-materials silicone 3D printing using UV-cured silicone technology (ACEO, Wacker Cheime, Germany] and moisture-cured silicone extrusion actuator [18].

Previously, 3D printing has focused mainly on the printing of bio-models for medical education and preoperative planning and trainings, with special cases of 3D printed titanium calcaneal, spinal and dental implants. To print medical models, implants or devices, one can first use the FDA-approved MIMICS software (Materialise, Belgium] to convert the DICOM files of the patients’ CT-MRI to the STL 3D printable format or an open-source Slice3r software for practice.

This new technology certainly opens up the gateway to rapidly 3D print various customizable medical silicone implants and devices for patients and fills the current gap in the AM industry, since the current AM technology have not involved direct 3D printing of medical silicone implants.

The current study has shown that by the precise control of flow rate, nozzle diameter, nozzle temperature and platform temperature, it is possible to accurately print a customized meniscal implant. The starting heat temperatures required for the resins are obtained from the calibration curves shown in the result section. These temperatures ensure adequate degree of gelation prior to extrusion and optimal degree of inter-laminar bonding with previously extruded layers. With the progression of printing, these temperatures require fine modulation with time to cater for different geometries and thicknesses of the end-products.

In addition, the univariate linear regression analysis showed that there is a close correlation between the accuracy and variability of both the lengths and widths of the 3D printed meniscus and both the nozzle and print bed temperatures. A higher optimal temperature not only reduces variability of both the printouts of lengths and widths of the meniscus but also improves the precision and accuracy of printouts.

The simulation results also reflect quite accurately the precise setting of experimental temperature of the heating block at 60 °C to produce a nozzle output of silicone resin at around 40 °C, the extrusion of which provides optimal consistency and lamination with the previous layers. In contrast to the body of the printed meniscus implant, the anterior and posterior horns of the 3D printed meniscus implant retained the highest temperatures of about 80 °C upon completion of printing, making these sites are not suitable for further manipulation or incorporation of micro-channels, drugs or cellular components.

To ascertain the functionality of 3D printed silicone meniscus, the compressive mechanical properties are compared to heat-molded silicone meniscus. The results confirmed that the 3D printed silicone meniscus has similar compressive mechanical properties as that of the heat-molded silicone meniscus. This shows that the new process developed has potential to replace the current heat molding process. 

Although several biodegradable and biocompatible scaffolds are available on the market to reconstruct the segmental meniscus defects of previous parts, these scaffolds still need to consider the bulk material properties, structure design and functional requirements and the fabrication process also should be reproducible and reliable. 3D silicone direct printing based on extrusion technique are compared with common fabrication technologies used in meniscal tissue engineering and are tabulated in Table 3.

## 5. Conclusions

A novel silicone 3D printer was successfully built for the direct 3D Printing of silicone meniscus implants which demonstrate mechanical properties as the conventionally heat-molded meniscus. The nozzle diameter, nozzle and bed temperatures were shown to be critical factors in determining the precision and accuracy of the lengths and widths of the meniscus. In this study, heated printer nozzle and meniscus implant designs were evaluated to determine the thermal distribution along the nozzle and across the meniscus implant by employing CFD and heat simulations using Solidworks.

Incorporating complex interior lattice or micro-channels and composite multi-material 3D silicone printing will be the future challenges in direct silicone 3D printing. Scaffold-based tissue regeneration based on AM technology is another promising approach to meniscus surgical treatment. In theory, the meniscal scaffold should provide appropriate biomechanical functions after implantation to shield cells from damaging compressive or tensile forces, maintain their shape integrity (without shrinkage, etc.), mechanical stability and strength on the defect area until enough host tissue was regenerated, produce mechanical stimuli to promote tissue regeneration.

Unique challenges present in silicone 3d printing are: (1) difficulty in the handling of silicone resins, (2) difficulty in printing multi-materials or different silicone resins, (3) finding suitable post-processing methods and 4) coming up with suitable standards in medical silicone 3D Printing.

Handling of silicone resins require meticulous even mixing to avoid trapping of air bubbles. Two-part resins are prone to disproportionate mixing and uneven curing. One-part resins are susceptible to moisture contamination and premature curing.

Different grades of silicone resins or different materials require different printing parameters and printing processes for optimal output. Consequently, modifications and additions to the 3D printers are necessary to achieve multi-grade silicones or multi-material printing.

Unlike post-processing methods in the 3D printing of other solid, liquid or powder substrates, silicone rubber products are highly susceptible to cuts, fissures, abrasions and lacerations while undergoing post-processing. Conventional processing methods for the above substrates cannot be applied to silicone printing. Therefore, this is a unique case where post-processing should be avoided as much as possible.

Currently no ASTM or similar standards was prescribed for medical silicone 3D printing. It is therefore imperative that one of the major areas of future works focus on the setting of standards in medical silicone 3D printing.

## Figures and Tables

**Table 1:** Ecoflex sample technical data (from safety data sheet provided by Smooth-On Inc).

	Mixed Viscosity (cp)	Specific Gravity (g/cc)	Specific Vol. (cu. in./lb.)	Pot Life (min)	Cure Time (h)	Shore Hardness	Tensile Strength (psi)	100% Modulus (psi)	Elongation at Break (%)	Die B Tear Strength (pli)	Shrinkage (in./in.)
Ecoflex 50	8000	1.07	25.9	18	3	00–50	315	12	980	50	<0.001
Ecoflex 30	3000	1.07	26.0	45	4	00–30	200	10	900	38	<0.001

**Table 2:** Univariate linear regression analysis to predict meniscus length and width using nozzle and print bed temperatures.

Independent Variables	Dependent Variables							
	Length (mm)				Width (mm)			
	Adjust R^2^	β	S.E.	*p*-Value	Adjust R^2^	β	S.E.	*p*-Value
Nozzle temperature (°C)	0.84	−0.01	0.002	*p* < 0.001	0.68	−0.01	0.003	0.002
Print bed temperature (°C)	0.62	−0.01	0.003	*p* < 0.001	0.64	−0.02	0.004	0.003

Adj: adjusted; S.E.: standard error.

**Table 3:** Comparison of direct silicone print with 3D scaffold for meniscus.

	3D Direct Printing Silicone Meniscus	3D Scaffold for Meniscus
**Material**	Silicone	Tissue-derived materials (e.g., periosteal tissue [19], small intestine submucosa (SIS) [20]), synthetic polymers (e.g., polycaprolactone (PCL), Hydrogels, etc.
**Fabrication method**	Direct silicone extrusion with heat cured Direct silicone extrusion with moisture cured UV cure (ACEO, Germany) Direct catalyst extrusion (Fripps, UK)	For sponge scaffold: leaching, gas foaming Fibrous scaffold: electrospinning, electrohydrodynamic jetting [32]
**Pros & cons**	(+) Biocompatible material approved by FDA (+) Minimal void inside (+) High strength and elongation (+) Relatively low price (−) Low resolution (−) limited capability to fabricate complex structures	(+) For sponge scaffold: high porosity benefit for cell proliferation, etc. (+) For fibrous scaffold: ability to fabricate nanofibrous architecture and complex structures; wild range of fiber diameter; (−) Using solvent can be toxic (−) Limited fabrication capabilities (−) Limited biochemical composition and biomechanical properties for implant

## Supplementary Materials

The following are available online at https://www.mdpi.com/2073-4360/12/5/1031/s1, Figure S1. (a) Model dimensions and (b) schematic of CFD design of heated nozzle. Figure S2. Changes in storage and loss modulus over time for (a and b) Eco30 and (c and d) Eco50 under heat curing of 40, 45, 55 and 65 °C. Figure S3. Bright field images of (a) topmost layer and (b) vertical cross-section of body of 3D printed silicone meniscus implant. Figure S4.. Comparison of thermal results using one-convection (above) and on-off (below) simulation. Table S1. Material Properties of Aluminum, Stainless Steel 304, Ecoflex Silicone. Table S2. Boundary conditions for printed silicone meniscus implant.) and on-off (below) simulation.
