# Supplementary material for: 3D Direct Printing of Silicone Meniscus Implant Using a Novel Heat-Cured Extrusion-Based Printer
Source: Polymers (Basel). 2020 May 1;12(5):1031. doi: 10.3390/polym12051031 (PMC7285157; doi:10.3390/polym12051031)
Supplement: Supplementary file 1 [file polymers-12-01031-s001.pdf]

Supporting Information

3D Direct Printing of Silicone Meniscus Implant Using a Novel Heat-Cured Extrusion Based Printer

Eric Luis [1], Houwen Matthew Pan [2], Swee Leong Sing [1], Ram Bajpai [3,4], Juha Song [2] and Wai Yee Yeong [1,*]

[1] Singapore Centre for 3D Printing, School of Mechanical and Aerospace Engineering, Nanyang Technological University, 50 Nanyang Avenue, Singapore 639798; G001@ntu.edu.sg (E.L.); slsing@ntu.edu.sg (S.L.S.); wyyeong@ntu.edu.sg (W.Y.Y.)

[2] School of Chemical and Biomedical Engineering, Nanyang Technological University, 70 Nanyang Avenue, Singapore 639798; matthew.pan@u.nus.edu (H.M.P.); songjuha@ntu.edu.sg (J.S.)

[3] Center for Population Health Sciences, Lee Kong Chian School of Medicine, Nanyang Technological University, 11 Mandalay Road, Singapore 308232; r.bajpai@keele.ac.uk (R.B.)

[4] School of Primary, Community and Social Care, Keele University, UK ST5 5BG; r.bajpai@keele.ac.uk (R.B.)

* Correspondence: wyyeong@ntu.edu.sg

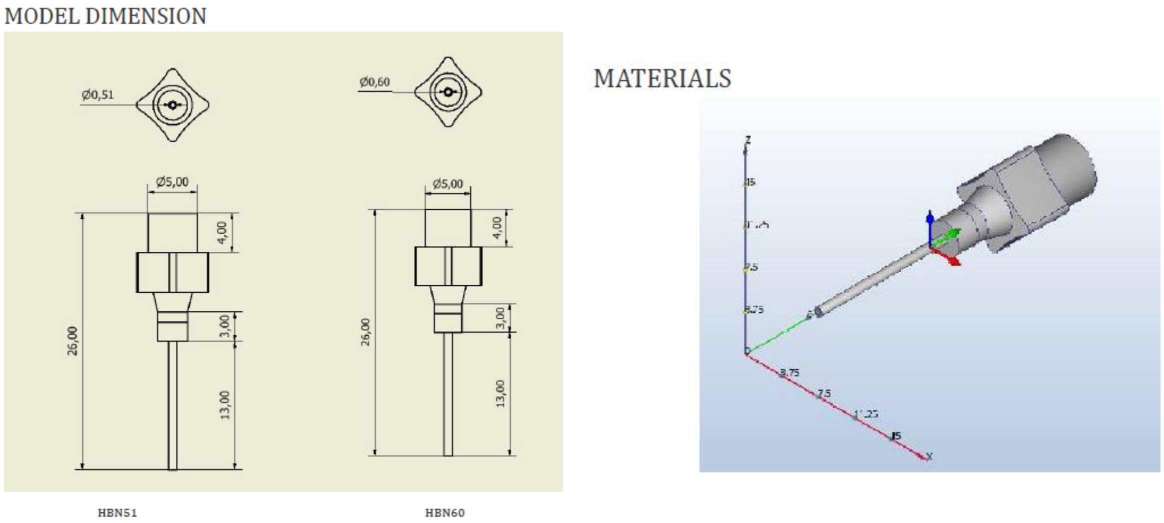

(**a**) (**b**)

Figure S1. (**a**) Model dimensions and (**b**) schematic of CFD design of heated nozzle.

Table S1. Material Properties of Aluminum, Stainless Steel 304, Ecoflex Silicone.

Name	Assigned To	Properties	
**Aluminium**	Heat block	X-Direction	204.0 W/m-K
		Y-Direction	Same as X-dir.
		Z-Direction	Same as X-dir.
		Density	2707.0 kg/m^3
		Specific Heat	896.0 J/kg-K
		Emissivity	0.2
		Transmissivity	0.0
		Electrical ressistivity	2.7e-08 ohm-m
		Wall roughness	0.0 meter
**Stainless Steel (304)**	Nozzle	X-Direction	Piecewise Linear
		Y-Direction	Same as X-dir.
		Z-Direction	Same as X-dir.
		Density	8.0 g/cm^3
		Specific Heat	0.5 J/g-K
		Emissivity	0.54
		Transmissivity	0.0
		Electrical ressistivity	7.2e-05 ohm-cm
		Wall roughness	0.0 meter
**Ecoflex 00-30**<br>**Ecoflex 00-50**	Medium	Density	1.07 g/cm^3
		Viscosity	8.0/3.0 Pa-s
		Conductivity	2.55 W/m-K
		Specific Heat	1.3 J/g-K
		Compressibility	2000.0 MPa
		Emissivity	1.0
		Wall roughness	0.0 millimeter
		Phase	Vapor Pressure

Table S2. Boundary conditions for printed silicone meniscus implant.

Layer	Perimeter (mm)	Time taken (s)
1	273	330
2	274	330
3	165.7	183
4	151.8	167
5	138.9	153
6	125.8	138
7	112.3	124
8	97.9	108
9	82.0	90
10	64.0	70
11	43.0	47
	Total Time	1740 (29 mins)

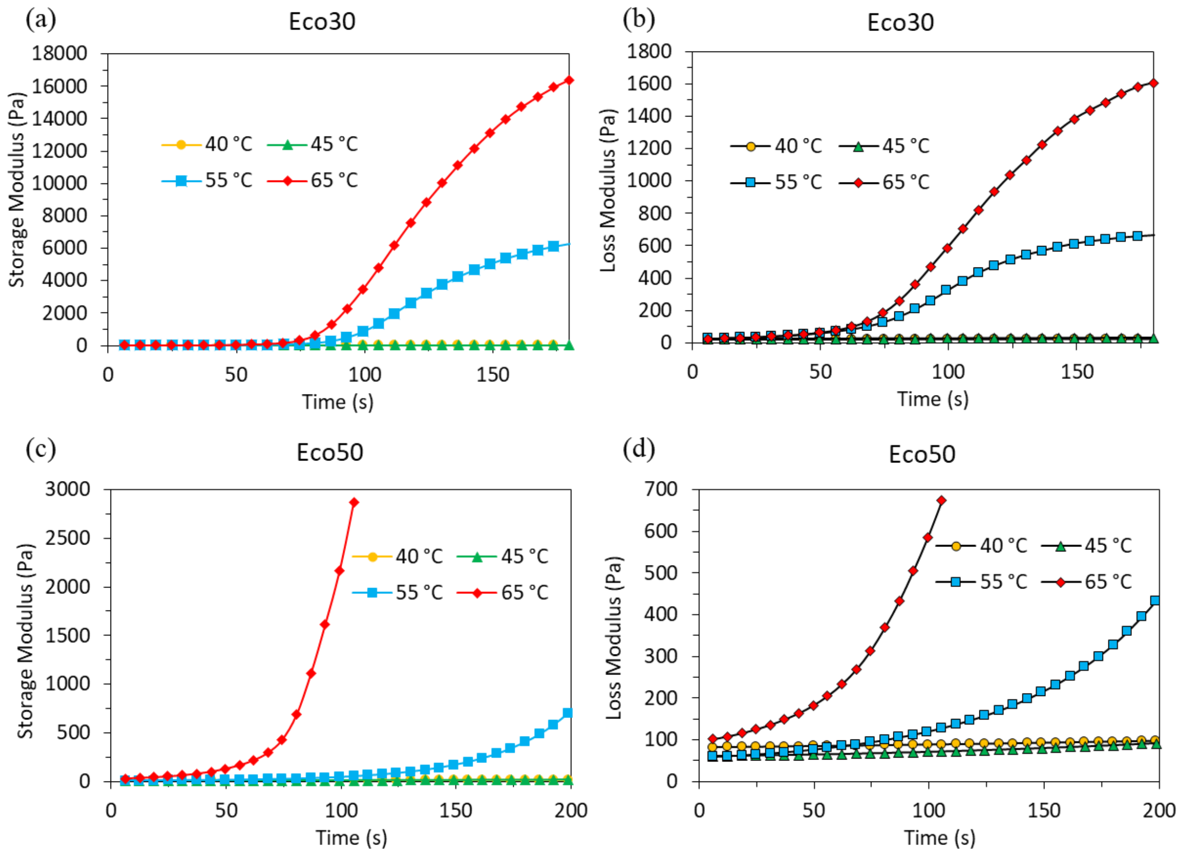

Figure S2. Change in storage and loss modulus over time for (a and b) Ecoflex30 and (c and d) Ecoflex50 under heat curing of 40, 45, 55, and 65 °C.

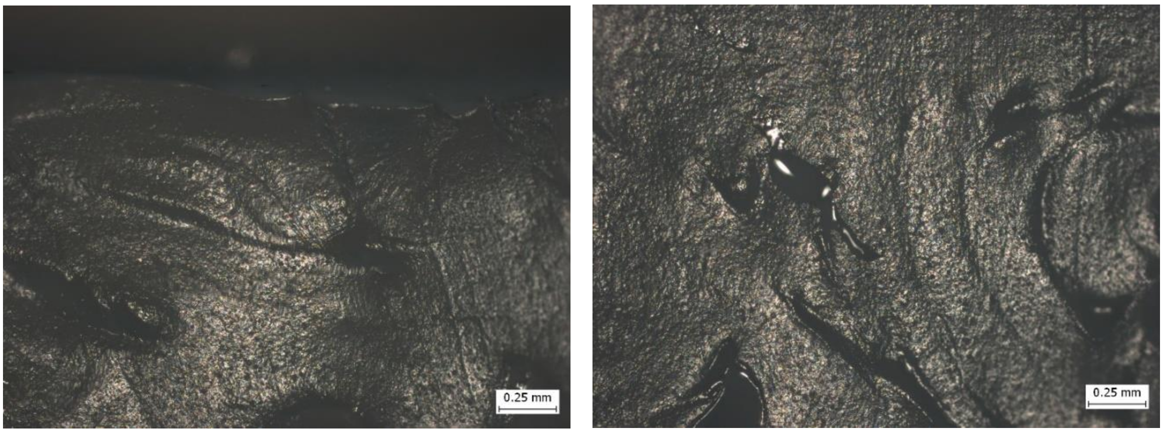

(**a**) (**b**)

Figure S3. Bright field images of (**a**) topmost layer and (**b**) vertical cross-section of body of 3D printed silicone meniscus implant.

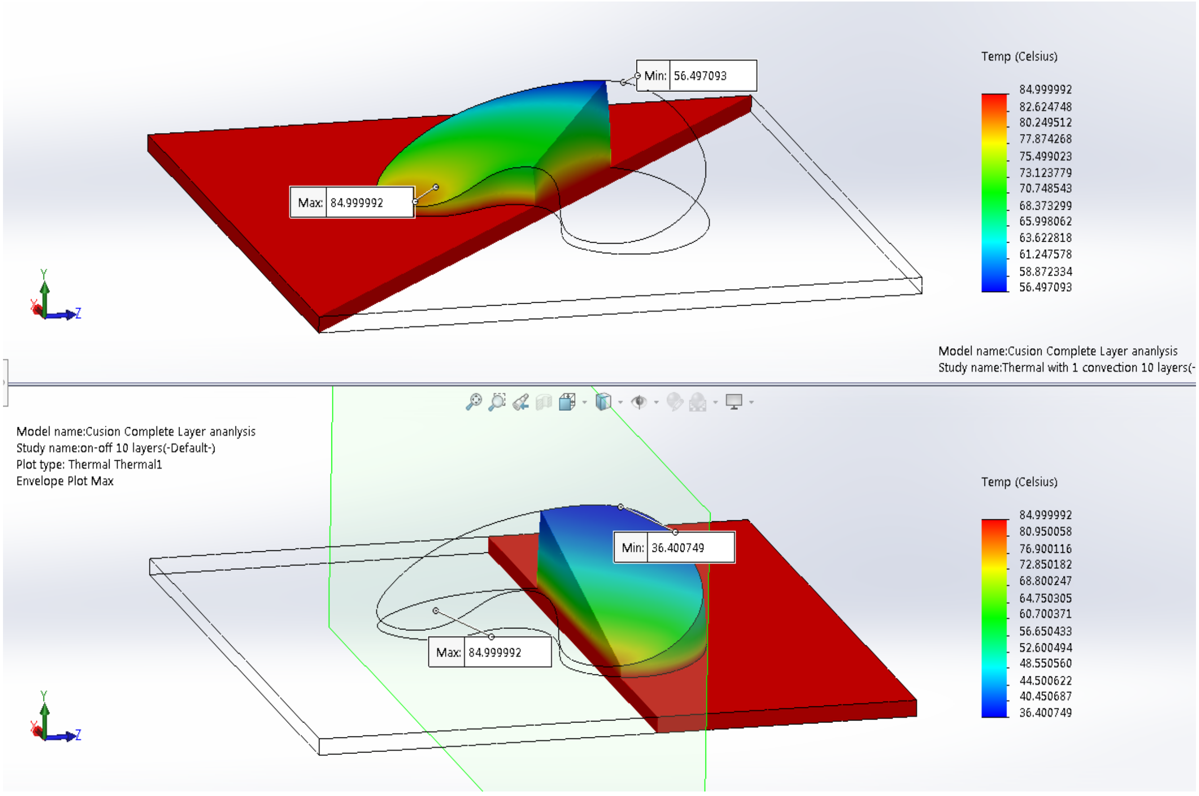

Figure S4. Comparison of thermal results using one-convection (above) and on-off (below) simulation.
